# Quantifying the Pro-Environmental Impacts of Telehealth Tobacco Treatment

**DOI:** 10.1089/tmr.2025.0019

**Published:** 2025-06-24

**Authors:** Sohayla Eldeeb, Amy Chieng, Cindy Tran Xu, Judith J. Prochaska

**Affiliations:** ^1^ Department of Medicine, Stanford Prevention Research Center, Stanford University, Palo Alto, California, USA.; ^2^ Health Education, Engagement and Promotion, Stanford Health Care, Menlo Park, California, USA.

**Keywords:** telehealth, greenhouse gas emission, patient travel reduction, environmental benefits, tobacco cessation treatment

## Abstract

**Introduction:**

Greenhouse gas (GHG) emissions trap in heat responsible for global warming. Stanford Tobacco Treatment Service uses telehealth for patient care.

**Methods:**

To quantify the environmental benefits of the clinic, data were abstracted from the electronic health record between March 17, 2020, and September 20, 2022. Round trip distances from address zip code to clinic were calculated to quantify GHG. Reductions in cigarettes from baseline to 24-month follow up were analyzed.

**Results:**

The sample of 556 patients averaged 2 sessions and 156.8 miles per round trip, saving 148.8 kg of GHG emissions per patient. Ninety-four patients had tobacco usage data at both timepoints; 83 (88%) used cigarettes and had an average decrease of 5.5 cigarettes per day.

**Conclusions:**

Applying findings to the 1,820 patients treated, the clinic has averted 271 metric tons of GHG travel emissions via telehealth; equivalent to 693,101 miles driven. Investing in telehealth tobacco cessation can prevent illnesses, health care expenditures, and environmental hazards.

## Introduction

During the COVID-19 pandemic, health system leaders and health care providers accelerated a shift to telehealth to support and sustain access to patient care.^1^ Telehealth, as defined by the Federal Communications Commission, “is similar to telemedicine but includes a wider variety of remote healthcare services beyond the doctor-patient relationship.”^2^ Key learnings from the pandemic-related increased shift to telehealth have provided opportunity to estimate telehealth’s benefits to the environment.

For example, between 2015 and 2019, at Pacific Northwestern Kaiser Permanente, an integrated health care system with >600,000 members, telehealth visits increased about 40% annually, and at the start of the pandemic in 2020, telehealth visits increased 108.5%, which notably reduced greenhouse gas (GHG) emissions by 51%.^3–5^ GHG emissions cover the earth’s surface with gases such as carbon dioxide (CO_2_), which trap in heat responsible for climate change and global warming. GHG emissions are primarily driven by activities such as transportation.^6–8^ Reduction of GHG emissions decreases the negative impacts of climate change such as cardiovascular disease, food and water borne illnesses, and weather related morbidity and mortality.^9^

Patient travel for health care emits CO_2_. According to calculations from the University of California, Davis (UC Davis) Health System, shifting 19,246 in-person consultations to telemedicine visits, in an 18-year period, would have reduced 1,969 metric tons of CO_2_ from patient travel,^10^ with each ton equal to 2,558 miles driven by a typical gasoline-powered passenger vehicle.^11^ Eliminating patient travel creates both considerable time and cost savings for the patients, allowing increased access to care by reducing these barriers.^12^

Tobacco use harms people and the planet.^13,14^ Cigarette production, combustion, and butt litter release toxic gases and particulates into the environment.^15^ Annually, starting with the cultivation of tobacco to the final disposal by smokers, 62.8 metric tons of CO_2_ are emitted into the air.^14^ In addition, the International Agency for Research on Cancer (IARC) has classified nine chemical constituents in cigarette smoke as carcinogens: benzene, cadmium, arsenic, nickel, chromium, 2-naphthyl-amine, vinyl chloride, 4-aminobiphenyl, and beryllium.^16^ For every 300 cigarettes produced, a tree is destroyed.^17^ Cigarette butt litter can be found in multiple publicly accessible environments.^18,19^ Based on California coastal cleanup day data from 1988–2023, cigarettes/cigarette filters are at the top, accounting for 35.34% of all trash items (N = 19,547,797).^20^ In 2020 alone, 190,042 cigarettes were found on California beaches and inland waterways.^21^

Receiving two Moonshot supplement awards from the National Cancer Institute, the Stanford Cancer Institute was one of 52 comprehensive cancer centers funded to integrate tobacco cessation treatment within cancer care services and to sustain the programs with matching commitments.^22^ The program offers tobacco treatment services through telehealth to Stanford Cancer Center patients who live in California. To date, since January 2019, of over 76,000 patients seen at the Stanford Cancer Center (SCC), 99% have been screened for tobacco use with 6% reporting current use. Our tobacco treatment specialist proactively calls all SCC patients identified as actively using tobacco and, to date, has reached 67% and engaged 42% into treatment (1,820 patients treated as of July 21, 2024). Our patient-reported outcomes obtained on 71.5% of patients treated indicate that 24% are successfully tobacco-free at 24 months. This estimate conservatively assumes that those lost to follow-up are not tobacco-free (i.e., missing = smoking)^23^ and our clinic tracks out to 24-month outcomes. Our rate of 24% tobacco-free is higher than the average abstinence rate of 20% reported for 20 cancer center tobacco treatment programs.^23^ Notably, the Stanford Tobacco Treatment Service has received local and national recognition for best-in-class outcomes with patient reach, engagement, and quit rates.^24–26^

## Objective

Extending beyond the personal health benefits of becoming tobacco-free, the current study aimed to quantify the pro-environmental impacts of the clinic. With telehealth tobacco treatment, we analyzed the decarbonization of patient travel and tobacco waste averted. We also wanted to look at barriers to access of care such as patient travel costs and distance. We believe this is the first analysis modeling the environmental benefits of a telehealth tobacco treatment service.

## Methods

The methodological approach centers around a proactive telehealth multidisciplinary treatment model designed to identify, engage, and provide treatment to SCC patients reporting tobacco use. A comprehensive suite of telehealth and digital health services is offered within this model, encompassing virtual medication consultations, behavioral counseling via virtual platforms such as Zoom, immersive virtual reality experiences, and text-to-quit services. Importantly, all these services are provided as covered benefits for SCC patients and their household, with interpreter services engaged as needed, to help reduce tobacco use or maintain abstinence if recently quit in the past 30 days.

**FIG. 1.**
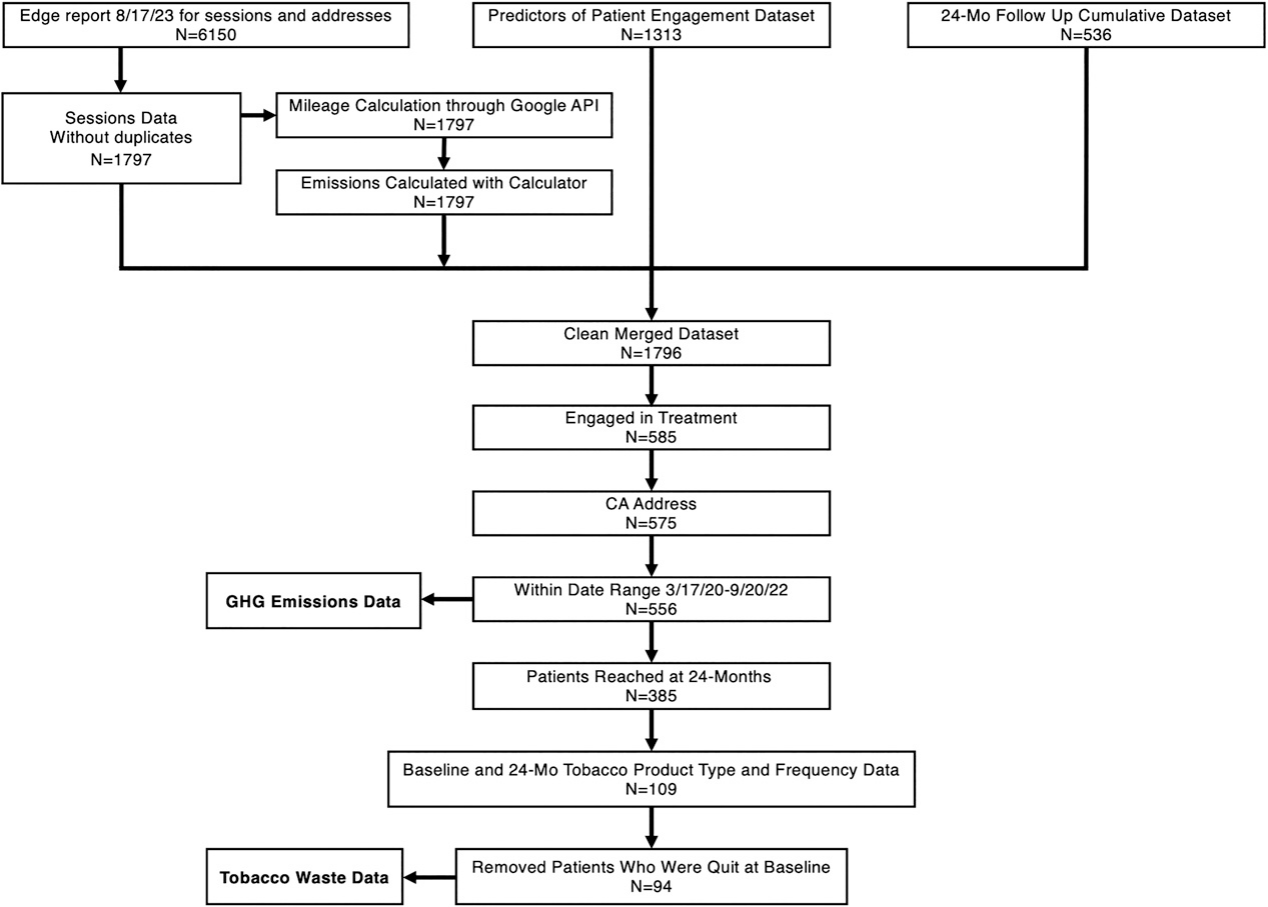
Consort diagram.

Integration of the Stanford Tobacco Treatment Service within cancer care started in January 2019^24^ with funding from the National Cancer Institute. The project included attention to service delivery and patient-reported outcomes as a quality improvement initiative (Stanford Institutional Review Board protocol 48420). Due to the quality improvement nature of the study, the need for informed consent was waived by Stanford Institutional Review Board. The data analyzed here were collected as part of the program’s quality improvement efforts. For the current analysis, data from the subset of patients with at least one documentation point during the COVID-19 pandemic from dates March 17, 2020, to September 20, 2022, were analyzed. This subset of patient cases was defined as part of a separate study on predictors of patient engagement with findings reported elsewhere.^27^ Data on these patients’ sessions, quit status, and addresses were obtained from the medical record, cleaned, merged, and consolidated (Fig. 1). Demographics of the final analytic sample of N = 556 for calculating GHG emissions data are presented in (Table 1).

**Table 1.** Baseline Demographics of the Sample, *N* = 556

Demographics	Mean (SD), range	*N* (%)
Age	59.3 (12.2), 26–91	
Sex		
Female		259 (46.6%)
Male		297 (53.4%)
Ethnicity		
Non-Hispanic/Latinx		462 (83.1%)
Hispanic/Latinx		76 (13.7%)
Unknown		18 (3.2%)
Race		
White		365 (65.6%)
Asian		41 (7.4%)
Black		32 (5.8%)
Native Hawaiian/Pacific Islander		9 (1.6%)
Alaska Native/American Indian		4 (0.7%)
Unknown		105 (18.9%)

To quantify the environmental benefits of telehealth, 6,150 rows of data obtained on August 17, 2023, were reviewed to code for the number of counseling and medication consultations conducted. Transportation miles avoided due to telehealth visits were calculated using round trip distances from patients’ addresses to the treating clinic at Stanford. These distances served as the basis for estimating associated GHG emissions, with emissions for various vehicle types derived from well-established statewide vehicle registration data.^28^ Of the options considered for estimating GHG emissions,^11,29^ a publicly available calculator was deemed most relevant as it utilized a life-cycle assessment to account for all emissions extracted from fuel vehicles—including the proportion of gasoline, hybrid, electric, and plug-in hybrids—from the state of California. The entire lifecycle of fuel production: extraction, processing, and transportation (well-to-tank), as well as its consumption and emissions during vehicle operation (tank-to-wheel) are considered in this computation.^30^ Furthermore, the calculator converts various GHG emissions into CO_2_ equivalents (CO_2_e) using global warming potentials (GWP). GWP is an index that reflects how much energy one ton of a specific GHG will absorb relative to 1 ton of CO_2_e, enabling comparisons of various emissions across sectors and gases. As an example, one round trip mile is calculated to be 0.47 kg of CO_2_e.

Due to the timing of when the data were acquired, documented addresses in the medical record may have changed since the patients’ time in the Tobacco Treatment Service. Patients now residing outside of California were excluded from the analyses so as not to inflate the GHG estimates. To determine travel distance and time to the tobacco treatment appointments, we utilized the Google Maps directions feature, centering on patients’ provided home addresses and integrated a Google Maps API to calculate travel distances to/from the tobacco cessation clinic. The route with the shortest travel time was selected.^29^ Emissions were converted into CO_2_e using the GHG emissions calculator.

Reduced tobacco consumption was quantified as cigarette equivalent reductions summed over time. The number of cigarettes smoked per day and/or abstinence status was assessed at baseline and 24 month via survey, phone call, or chart review. To aid with collection of these outcomes, clinical staff kept track of contact points and notes for each assessment period on password-protected Excel spreadsheets. As part of the coding process, qualitative notes within the 24-month assessment period were reviewed (N = 536) and coded manually to describe baseline and 24-month tobacco product usage with prioritization of combustibles (smoking products over nonsmoking products), frequency of use, and quit status. Combustible tobacco products were more commonly used and for simplicity of coding, one tobacco product per patient was coded. Change over time (calculated as *24-month usage*—*baseline usage*) was calculated for patients with both baseline and 24-month outcomes available on the same product type. In addition, at baseline, 15 out of the 109 patients who were quit were provided cessation support to maintain abstinence: 14 smoked cigarettes and 1 chewed tobacco. These patients were excluded from the tobacco waste analyses since they already quit on intake.

Patient cost savings were calculated based on transportation miles saved via telehealth and tobacco reduction. Miles saved were based on California recent gas prices^31^ and average miles per gallon.^32^ Tobacco reduction savings were extrapolated from retail costs in California.^33,34^

## Results

The analytic patient sample averaged 59.3 years of age (SD of 12.2), 83.1% were non-Hispanic/Latinx (83.1%), and 65.5% were White. Further demographic details are described in (Table 1).

### GHG emissions averted

In the sample of 556 patients, round trip distance averted via telehealth per patient was an average of 156.83 miles per session and patients averaged 2.02 sessions. By delivering treatment via telehealth, an average of 73.68 kg GHG emissions were averted per session and 148.83 kg GHG per patient (Table 2).

**Table 2.** Sessions per Patient and Single Session Round Trip in Miles and Emissions (*N* = 556)

	Mean	SD	Median	IQR	Range
Sessions including intake	2.02	3.20	1.00	1.00 to 2.00	1.00 to 42.00
Single session round trip miles	156.83	145.27	101.71	47.48 to 245.23	2.95 to 969.13
Total GHG emissions round trip^a^	73.68	68.25	47.79	22.31 to 115.21	1.39 to 455.32

^a^ Calculated with Dr. Melissa Frick’s calculator.^30^

GHG, greenhouse gas.

### Tobacco waste averted

The data on tobacco waste averted at 24 months (N = 94 patients) include 83 patients (88.3% of sample) smoking cigarettes with 39.4% (37/94) quit, 9 (9.6% of sample) using chew tobacco with 5.3% (5/94) quit, 1 smoking cigars (quit), and 1 using vapes (quit). Among those who had not quit, an additional 14 people smoking cigarettes had reduced their use by 50% or more.

Among those smoking cigarettes, the mean per patient change from baseline to the 24-month follow-up was a decrease of 5.54 cigarettes per day (SD = 7.5; median = −4.0 [IQR = −10.0, −0.14]). Among those using chew tobacco, average per patient change from baseline to the 24-month follow-up was a decrease of 0.55 cans per day (SD = 0.68; median= −0.29 [IQR = −1.00, −0.10]). See Table 3.

**Table 3.** Per Patient Change in Use per Day from Baseline to 24 Months and Estimated Tobacco Waste Reduced over 2 Years’ Time for the Analytic Sample (*N* = 94)

Product (# of patients)	Mean	SD	Median	IQR	Range	Estimated tobacco waste reduced^a^
Cigarettes (*n* = 83)	−5.54	7.47	−4.00	−10.0 to −0.14	−40.00 to 10.00	335,668.60
Chew (*n* = 9)	−0.55	0.68	−0.29	−1.00 to −0.10	−2.00 to 0.17	3,613.50
Cigars (*n* = 1)	−10.00	N/A	N/A	N/A	N/A	7,300.00
Vape (*n* = 1)	−1.00	N/A	N/A	N/A	N/A	730.00

^a^ Estimated tobacco waste reduced calculated as n (# of participants) × mean reduction × 365 × 2.

Zafeiridou, Hopkinson, and Voulvoulis (2018) noted a typical smoked cigarette had a climate change impact of 14 g of GHG.^14^ Applying this value, a decrease of 5.54 cigarettes per day reduces 77.6 g of GHG daily, meaning 28.3 kg of GHG were averted in a year by an average patient; 4,699.4 kg (4.7 metric tons) of GHG were averted by the 83 patients in this dataset in a span of 2 years. There are no known conversions to CO_2_e to calculate the impact of reducing tobacco chew, cigars, or vapes.

### Cost savings for patients

In calculating the GHG emissions, we assumed each patient would have taken a car. As of July 22, 2024, California gas prices average U.S. $4.37 per gallon. The average gas-powered car gets 24 miles per gallon.^32^ By treating tobacco via telehealth, the average cost savings of gas per patient in the analytic sample for a single round trip was calculated as U.S. $28.56.

The average cost of a pack of cigarettes in the San Francisco Bay Area is U.S. $9.63 without tax, and U.S. $12.50 with tax.^33^ The average cost of chew tobacco in California is U.S. $7.00 per can.^34^ Over the 2-year follow-up, the sample averaged a reduction of 5.54 cigarettes per day, saving U.S. $3.46 per day; and a 55% reduction in chew tobacco, saving U.S. $3.85 per day, which in a year, would be savings of U.S. $1,263 and U.S. $1,405 per patient, respectively.

## Discussion

The current study undertook a multicomponent approach to quantify GHG emission reductions from both averted patient travel and reduced tobacco consumption. With added value for patients, analyses also quantified patient financial savings in reduced gas and tobacco costs.

On average, a telehealth patient session averted 73.7 kg of GHG by commuting mileage saved. The sample averaged 2 sessions, with one as many as 42 sessions (445.3 kg of GHG). Estimated tobacco waste averted by the analytic sample in a 2-year period are 335,668.6 cigarettes and 3,613.5 cans of chewing tobacco. The GHG emissions reduced among those who use cigarettes is estimated to be 4,699.4 kg of GHG in a span of 2 years; equivalent to 12,019 miles driven.^11^ Total average savings among the analytic sample from travel is U.S. $32,076, from cigarettes is U.S. $209,658 in a 2-year period, and from chew tobacco is U.S. $25,290 in a 2-year period.

Treating tobacco addiction as a chronic care condition necessitates repeated contacts.^35^ Telemedicine provides a feasible, convenient, and sustainable approach to cutting emissions without sacrificing patient care. Realistically, given the added travel logistics, had the service not been offered via telehealth, the number of sessions completed likely would have dropped along with the quit rates. Telemedicine servicing allows accessible health care for patients who face logistical or financial barriers to in-person care while mitigating the immense emissions associated with consistent appointments.

Applying the calculation for travel emissions averted to the entire 1,820 patients in treatment to date would be 271 metric tons of GHG averted in travel, which is equivalent to 693,101 miles driven and is over US $100,000 saved in gas costs.^11^

The UC Davis Health System’s study on miles saved from outpatient specialty sessions averaged 278 miles (SD 228 miles) per session, with estimated total emissions of 1,969 metric tons of GHG.^10^ The patients in the Stanford Tobacco Treatment Service, on average, have shorter round trips per session. We anticipate differences due to the miles driven across longer distances for the UC Davis patients. In addition, the calculations used for the overall GHG emissions averted for our study utilized a California-specific vehicle registration statistics, whereas the UC Davis study referenced a 2008 national vehicle consumption report.^10^

Most (88%) of the analytic sample smoked cigarettes, while national data indicate that 62% of the 46 million U.S. adults who use tobacco products smoke cigarettes (28.3 million people).^36^ The higher proportion of patients using tobacco products who smoke cigarettes is likely due to the sample being defined as engaging in cessation treatment supports. Newer products and patients who use smokeless tobacco products, such as nicotine pouches, or use vapes may be less likely to be identified on screening and/or engage in cessation services. Given rising use,^36,37^ health care providers should anticipate supporting patients in quitting vapes, which not only contain toxic compounds but also contribute to chemical, metal, and plastic waste.^13^

Cigarette butt litter can be found in multiple publicly accessible settings such as coasts, schools, and transportation stops.^18–20^ Locally, California coastal data are collected annually, with 108,869 cigarette filters found in 2023.^20^ In a 2018 environmental scan on Stanford’s campus, 596 discarded butts were collected over a 4-hour period in 1 day, and 296 butts in the forest and lake area were collected in a 2-hour period in 1 day.^19^ In treating patients who use tobacco, cigarette butt litter should reduce.

Leveraging telehealth for treating tobacco use in patients receiving cancer care can yield significant financial savings for patients in terms of tobacco purchases and travel for treatment. Further research may be done to explore cost-savings for health care systems with outpatient transportation services. The patient population served here was mostly middle aged to older adults. While the digital divide is narrowing,^38^ our team continues to invest in and support patient access to telehealth tobacco cessation efforts, allowing services to reach a wider range of patients who may have been excluded due to technological limitations. By shifting toward telehealth, patients will be able to minimize missed appointments and focus on consistent engagement to support overall health while reducing GHG emissions.

### Study limitations and strengths

Due to data gaps, we were conservative in our methods and relied upon well-informed assumptions. Counseling sessions were undercounted due to missing data in EHR flowsheets and incomplete patient information prior to the service’s EHR transition on July 26, 2020. Addresses in the EHR can change over time. Patients that moved away may affect the emissions calculations by overestimating travel emissions, while patients that moved closer may have led to underestimates. Patients with out-of-state addresses were not included in these analyses. Staff relied on counselor notes in the flowsheet during the coding process. To simplify the coding process, multiple tobacco use was limited to one product, and cigarettes were prioritized. Product switches were often to another combustible tobacco product and were not considered as quit. Notably, this is the first project to quantify the environmental impacts from telehealth tobacco cessation treatment.

Our evaluation is innovative in studying telehealth and tobacco treatment models to support patients with cancer to help alleviate GHG emissions from transportation and cigarette production and use. Our findings speak to the experience at single large cancer center in the state of California. To date, few studies have examined the impacts of telemedicine on GHG emissions, and further study is warranted.^39^

## Conclusions

Litter from tobacco use is an environmental hazard,^13–15,18^ and measuring environmental impacts from treatment services are important for supporting sustainable health care. Investing in telehealth tobacco cessation programs can yield substantial long-term benefits by averting tobacco-related illnesses, associated expenditures, and environmental hazards. This quality improvement initiative lends support to sustainable health care delivery models that mitigate the environmental impact of patient travel and treatment. Future evaluations would be strengthened by exploring long-term health and environmental outcomes associated with telehealth and reduction in tobacco use. Broadly, successful telehealth programs with measures of health and environmental impact may serve as a model for public health efforts.

As the world grapples with the dual challenges of health care provision and environmental sustainability, the integration of telehealth services stands out as a pivotal solution for enhancing patient accessibility, improving health outcomes, and simultaneously contributing to environmental conservation by reducing unnecessary travel and associated emissions. It is imperative for stakeholders across the health care spectrum to prioritize sustainability, innovation, and embrace telehealth as a cornerstone of modern health care delivery. Collaborative efforts, local and global, to increase telehealth and minimize the digital divide can pave the way for healthier communities for generations to come.

## Data Sharing Statement

Deidentified data that support the findings of this study are available upon reasonable request from the corresponding author J.J.P.

## Abbreviations Used

CA: California
CO_2_: Carbon dioxide
CO_2e_: Carbon dioxide equivalents
COVID-19: Coronavirus Disease 2019
EHR: Electronic health record
GHG: Greenhouse gases
GWP: Global warming potentials
IARC: International Agency for Research on Cancer
SCC: Stanford Cancer Center
UC Davis: University of California, Davis
U.S.: United States
